# Essential Oils: Useful Tools in Storage-Pest Management

**DOI:** 10.3390/plants11223077

**Published:** 2022-11-13

**Authors:** Ľudovít Cagáň, Miroslava Apacsová Fusková, Daniela Hlávková, Oxana Skoková Habuštová

**Affiliations:** 1 Department of Plant Protection, Slovak University of Agriculture, 949 76 Nitra, Slovakia; 2 National Agricultural and Food Centre, Research Institute of Plant Production, 921 01 Piešťany, Slovakia; 3 Biology Centre, Czech Academy of Sciences, Institute of Entomology, 370 05 České Budějovice, Czech Republic; 4 Faculty of Science, University of South Bohemia, Branišovská 31a, 370 05 České Budějovice, Czech Republic

**Keywords:** insect repellents, DEET, 2-undecanone, essential oils, *Tribolium confusum*, *Tenebrio molitor*, *Acanthoscelides obtectus*

## Abstract

This study aimed to verify the level of repellent and mortality effect of two chemical substances (DEET and 2-undecanone) and seven essential oils (EOs), *Allium sativum*, *Artemisia annua*, *Ocimum basilicum*, *Lavandula angustifolia*, *Eucalyptus globulus*, *Pinus sylvestris*, and *Curcuma longa*. The storage pests *Tribolium confusum*, *Tenebrio molitor*, and *Acanthoscelides obtectus* were exposed to various concentrations in an olfactometer-and-mortality test. The effects were recorded 24–48–72 h after the treatments were applied. *A. sativum*, *E. globulus*, and *L. augustifolia* were found to have significant repellence effects. A substantial lethal effect was observed for *A. sativum*, *E. globulus*, and *O. basilicum*. We also found that even if the most efficient EOs were diluted to low concentrations, they still produced repellent and mortality effects. The presented results indicate that *A. sativum* and *O. basilicum* were the most effective against *T. confusum* and *T. molitor;* simultaneously, *L. angustifolia* and *C. longa* showed high activity against *A. obtectus*. All of these efficient EOs could be applied as effective bio-control agents in various stored conditions.

## 1. Introduction

There are more than 20,000 species of field and storage pests, which destroy roughly one-third of the world’s food production annually [1]. Furthermore, storage insects can cause significant crop-production losses, ranging from 9% to 20%, or even higher, in developing countries [2]. The most common are (1) the yellow mealworm beetle, *Tenebrio molitor* (Linnaeus, 1758) (Coleoptera: Tenebrionidae)—a species of “darkling beetle” [3]; (2) the confused flour beetle, *Tribolium confusum* (Jacquelin du Val, 1863) (Coleoptera: Tenebrionidae)—a cosmopolitan pest occurring in many flour mills, warehouses, and grocery stores, whose ability to move quickly between patches, makes it one of the major pests in stored products [4]; and (3) the bean weevil *Acanthoscelides obtectus* (Say, 1831) (Coleoptera: Bruchidae), a Mesoamerican beetle known as a serious post-harvest and field pest of the common bean (*Phaseolus vulgaris* L.) [5]. 

Contemporary storage pests are eradicated by using fumigants. Considering the increasing application of commercially available synthetic fumigants and their negative impact on human health and the environment [6], together with pests’ resistance to them, finding a new alternative to toxic fumigants that is safe for the environment and non-target animals is critical [7].

Essential oils (EOs) are produced by 17,500 aromatic species of higher plants belonging primarily to the families of Myrtaceae, Lauraceae, Lamiaceae, and Asteraceae [8]. For example, EOs are extracted from eucalyptus leaves (*Eucalyptus globulus*) [9], garlic (*Allium sativum*) [10], basil (*Ocimum basilicum*) [11], turmeric (*Curcuma longa*) [12], lavender (*Lavandula angustifolia*) [13], Scot’s pine tree (*Pinus sylvestris*) [14], and sweet wormwood (*Artemisia annua*) [11]. 

Essential oils are complex mixtures of volatile secondary metabolites produced by aromatic plants; they are obtained by distillation and steam distillation. These volatile molecules include predominantly terpenes, monoterpenes, and sesquiterpenes, respectively. However, their chemical structures might be modified by oxidation, rearranging the skeletons, or, rarely, during biosynthesis. In this case, the specific subunits (i.e., alcohols, aldehydes, phenols, ethers, ketones) or functional groups (i.e., sulphur or nitrogen) are attached or integrated into the structure. These chemicals are known as terpenoids. Each EO comprises only two to five main components, which constitute up to 60% of the oil [15,16,17,18]. The most frequently mentioned substances in EOs are β-caryophyllene (CAS 87-44-5), D-limonene (CAS 138-86-3), α-pinene (CAS 80-56-8), α-terpineol (CAS 98-55-5), δ-cadinene (CAS 483-76-1), α-humulene (CAS 6753-98-6), and *p*-cymene (CAS 99-87-6) [17]. The toxicity of various EO constituents is well described [18].

No proven natural fumigants are currently employed to combat pests attacking grains, dry-stored food, or other agricultural products. Some of the fumigants that are frequently used to protect stored commodities are phosphine, methyl bromide, and DDVP (2,2-dichlorovinyl dimethyl phosphate) [19]. On the other hand, the most popular and effective natural-product-based insect-repellent substances are DEET (N,N-diethyl-3-methylbenzamide) and 2-undecanone (methyl nonyl ketone). These essences protect against stinging insects. Interestingly, 2-undecanone is a natural compound found in soybean, palm-kernel oil and a garden rue *Ruta graveolens* [20,21,22]. Unfortunately, the suitability of its use against storage pests is unknown. Furthermore, essential oils (EOs) appear to be feasible tools in pest management. This is especially due to their fungicidal, insecticidal, and herbicidal effects on pests. 

From the current vantage point, the use of EOs is problematic in many aspects. Some arise from chemical characteristics, such as their volatility and high degradation rate. Specifically, the oily and volatile qualities and particular chemical compositions may change the food-packaging features and alter the material resistance. Therefore, only some specific EOs can be included in food packaging despite their environmentally friendly and non-toxic qualities [23]. 

The biological activities of EOs are also problematic as they depend on the chemical composition. This is affected by factors such as the extraction method, plant phenology, timing of harvest season, location and age of the plant, soil condition, and many more environmental factors. Thus, each plant’s combination of chemicals is unique [24]. Unstandardized amounts of EOs might hinder their use in organic farming, even though their potential is immense. Other problems lead to costs connected with approval and registration processes. However, there discussions are currently underway to create a streamlined registration process for low-risk products (i.e., products that must not be toxic to non-target organisms and have a low soil persistency) [25]. The use of EOs is constrained for a variety of reasons, including a lack of specific regulations, a lack of knowledge about their effectiveness and negative effects, and their economic, consumer, and environmental impact, despite efforts to register EOs as flavourings and classify them as “Generally Recognized as Safe” (GRAS) substances [23]. 

According to Directive 128/2009 [26], the EU member states are obliged to reduce the use of synthetic pesticides and promote alternative means of pest control. Therefore, considerable efforts have been expended over recent years to use plant-based products (botanicals) for insect control [27], including stored product pests.

Based on the previously mentioned considerations of the necessity of new bioactive pest-control compounds of botanical origin, our work’s objective was to investigate and compare chemical compounds of the most frequently used essential oils and their repellence against *T. confusum*, *T. molitor*, and *A. obtectus* and to choose the most effective.

## 2. Results

### 2.1. Gas Chromatography–Mass Spectrometry

The chemical profiles of the EOs consisted of three to four dominant compounds and several lesser compounds with different positions. A gas chromatography–mass spectrometry analysis (GC–MS) of the selected essential oils revealed 23 compounds in *O. basilicum*, 57 in *A. sativum*, 82 in *C. longa*, 9 in *E. globulus*, 63 in *L. angustifolia*, 33 in *P. sylvestris*, and 14 in *A. annua*. Monoterpenes, organic disulfides, or terpenes accounted for the majority of the constituents. Some components of EOs, such as eucalyptol, carveol, o-cymene, and D-limonene, were detected in more than one species. Other components, such as citral for *O. basilicum*, eucalyptol for *E. globulus*, and tumerone for *C. longa*, were found to be species-specific. Detailed results can be found in Supplementary Table S1.

In brief:

- ***Ocinum basilicum***: trans-dihydrocarveol (17.81%, CAS 18383-51-2) and α -bisabolene (2.3%, CAS 17627-44-0).
- ***Allium sativum*:** diallyl disulphide (25.8%, CAS 2179-57-9), diallyl sulfide (11.66%, CAS 592-88-1), diallyl tetradulfide (5.15%, CAS 2444-49-7), and diallyl trisulfide (2.7%, CAS 2050-87-5).
- ***Artemisia annua***: eucalyptol (12.13%, CAS 470-82-6), trans-dihydrocarveol (7.11%, CAS 18383-51-2), and artemisia ketone (6.6%, CAS 546-49-6).
- ***Eucalyptus globulus***: eucalyptol (85.79%, CAS 470-82-6), alloocimene (7.55%, CAS 3016-19-1), and gamma-terpinene (3.67%, CAS 99-85-4).
- ***Pinus sylvestris***: norbornene (11.75%, CAS 497-32-5), D-limonene (11.65%, CAS 138-86-3), and β-pinene (5.03%, CAS 127-91-3).
- ***Lavender angustifolia***: β-ocimene (5.24%, CAS 3338-55-4), caryophyllene (4.11%, CAS 87-44-5), and lavandulyl acetate (3.24%, CAS 20777-39-3).
- ***Curcuma longa***: tumerone (26.15%, CAS 180315-67-7), quinoxaline (6.93%, CAS 7153-23-3).

### 2.2. Olfactometer Study 

**Figure 1:**
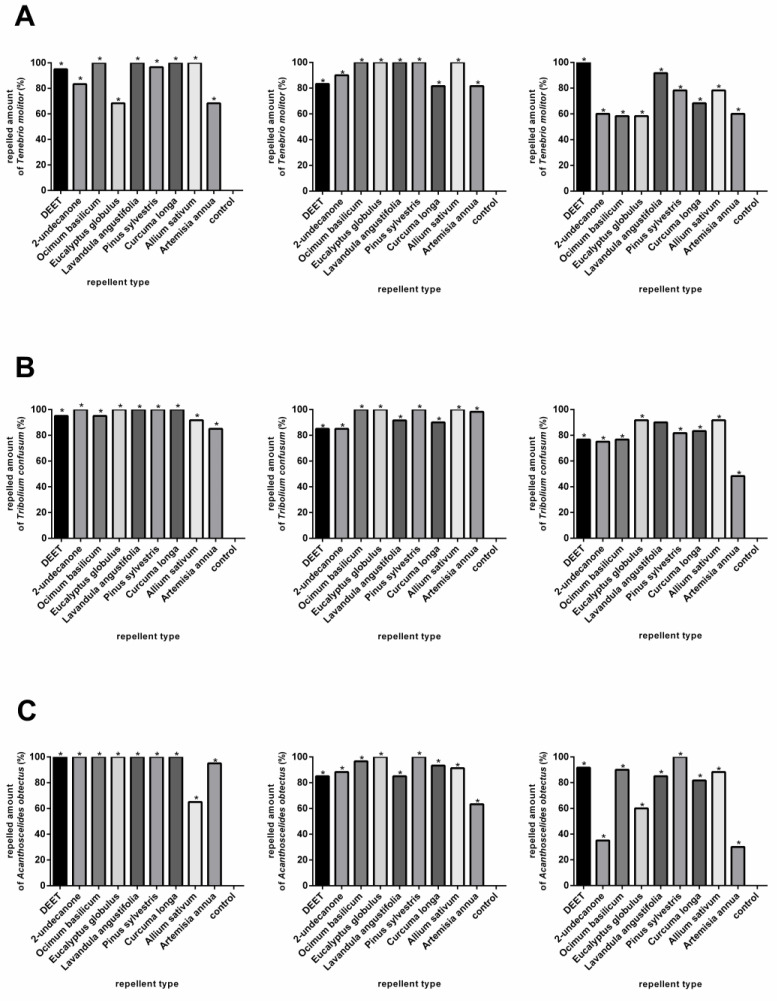
The reactions of *Tenebrio molitor* (**A**), *Tribolium confusum* (**B**), and *Acanthoscelides obtectus* (**C**) to chemical products (DEET and 2-undecanone) and essential oils (*Ocimum basilicum*, *Eucalyptus globulus, Lavandula angustifolia, Pinus sylvestris, Curcuma oblonga, Alium sativum*, and *Artemisia annua*) in comparison to control group. Significant differences are marked with an asterisk (*). The order of the graphs determines the concentrations used in the experiments (1%; 0.1% and 0.01%). Means of attracted individuals are pictured.

In the case of *Tenebrio molitor*, all the chemical substances and EOs were repellent at concentrations of 1% and 0.1%. At a concentration of 0.01%, only the EOs of *A. sativum*, *L. augustifolia*, *P. sylvestris*, and the chemical DEET caused significant repellence compared with the control variant (Figure 1A, Supplementary Table S2).

The *Tenebrio confusum* showed a significant response to all repellent substances at all concentrations except *A. annua* at a concentration of 0.01% (Figure 1B, Supplementary Table S2). At a concentration of 0.1%, five EOs (*A. sativum*, *A. annua*, *E. globulus*, *O. basilicum*, *P. sylvestris*) showed significantly higher repellence than the chemical substances. The highest repellence was achieved at a concentration of 0.01% and was observed only in the EOs of *A. sativum* and *E. globulus* (Figure 1B, Supplementary Table S2). The repellent tests with *A. obtectus* showed that all the compound substances except the EO from *A. annua* were significantly effective at concentrations of 1% and 0.1%. The 2-undecanone and EOs from the *A. annua* and *E. globulus* showed little or no repellent activity at concentrations of 0.01% (Figure 1C, Supplementary Table S2). As shown in Figure 1A–C, the essential oils of *A. sativum*, *E. globulus*, and *L. augustifolia* were very effective against all three storage pests.

### 2.3. Mortality Study

*Tenebrio molitor*

**Figure 2:**
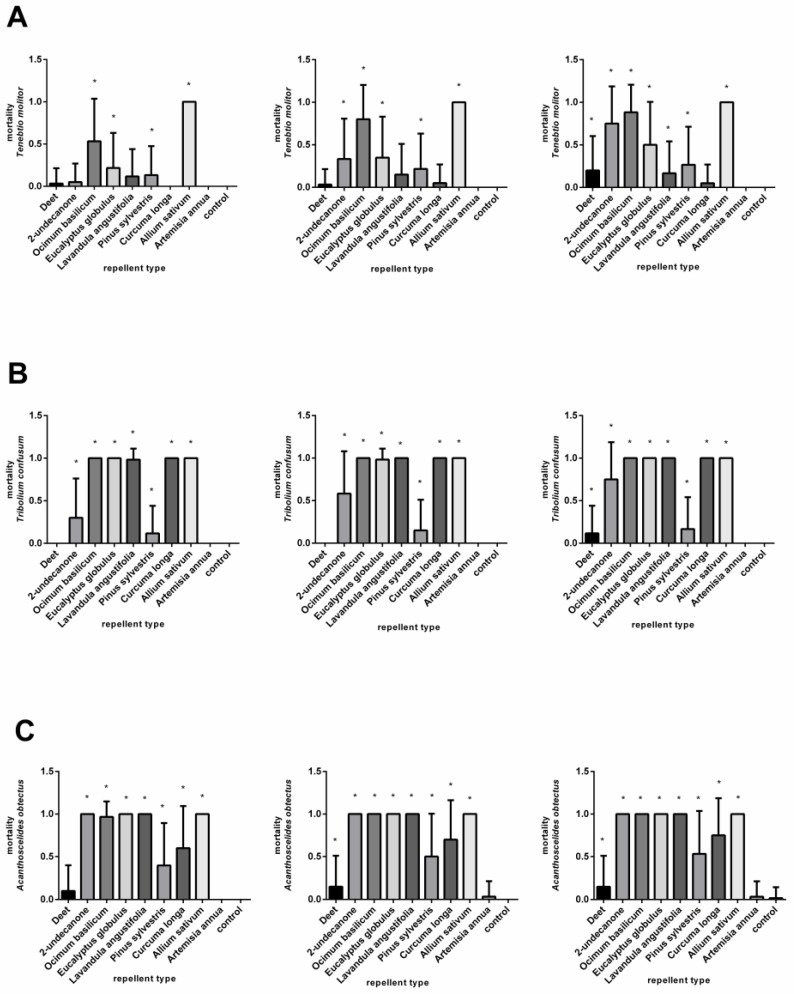
Mortality of *Tenebrio molitor* (**A**), *Tribolium confusum* (**B**), and *Acanthoscelides obtectus* (**C**) after using chemical products (DEET and 2-undecanone) and essential oils (*Ocimum basilicum*, *Eucalyptus globulus, Lavandula angustifolia, Pinus sylvestris, Curcuma oblonga, Alium sativum*, and *Artemisia annua*) at a concentration of 3.7 µL/cm^2^ in comparison to control group. Significant differences are marked with an asterisk (*). The order of the graphs determines the incubation time (24, 48, and 72 h) after application. Each column shows mean of dead individuals and SD.

The mortality effects of the various compounds on the adult *T. molitor* after 24, 48, and 72 h are shown in Figure 2A and in Supplementary Table S3. At a concentration of 3.7 μL/cm^2^, all the substances except for the DEET and *A. annua* showed significant mortality. However, the highest mortality was obtained only after the application of the EOs from the *A. sativum* and *O. basilicum*.

**Figure 3:**
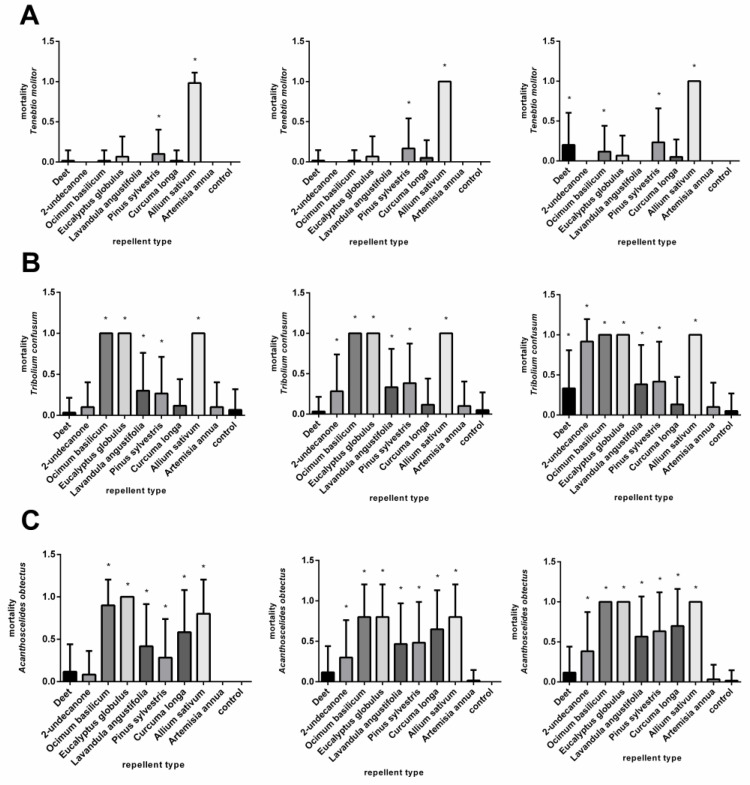
Mortality of *Tenebrio molitor* (**A**), *Tribolium confusum* (**B**), and *Acanthoscelides obtectus* (**C**) after using chemical products (DEET and 2-undecanone) and essential oils (*Ocimum basilicum*, *Eucalyptus globulus, Lavandula angustifolia, Pinus sylvestris, Curcuma oblonga, Alium sativum,* and *Artemisia annua*) at a concentration of 0.37 µL/cm^2^ in comparison to control group. Significant differences are marked with an asterisk (*). The order of the graphs determines the incubation time (24, 48, and 72 h) after application. Each column shows mean of dead individuals and SD.

**Figure 4:**
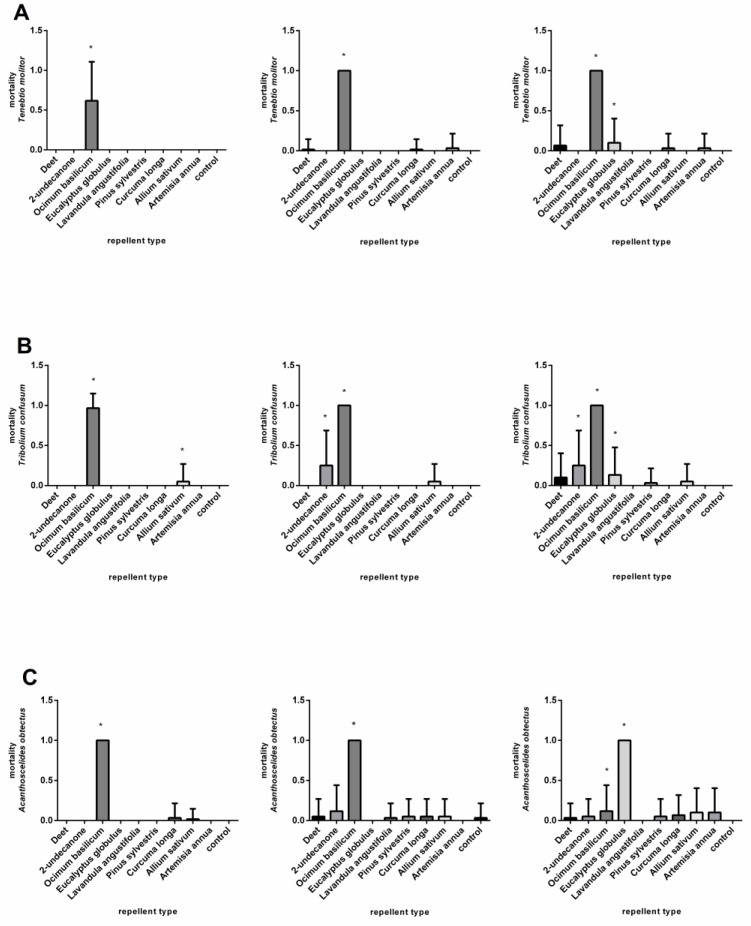
Mortality of *Tenebrio molitor* (**A**), *Tribolium confusum* (**B**) and *Acanthoscelides obtectus* (**C**) after using chemical products (DEET and 2-undecanone) and essential oils (*Ocimum basilicum*, *Eucalyptus globulus, Lavandula angustifolia, Pinus sylvestris, Curcuma oblonga, Alium sativum*, and *Artemisia annua*) at a concentration of 0.07 µL/cm^2^ in comparison to control group. Significant differences are marked with an asterisk (*). The order of the graphs determines the incubation time (24, 48, and 72 h) after application. Each column shows mean of dead individuals and SD.

The application of the *A. sativum* EO at a medium concentration, 0.37 μL/cm^2^, resulted in death within 24 h for all the animals tested. A significant mortality effect was observed only after the application of DEET, *P. sylvestris*, and *O. basilicum* EOs 72 h after treatment (Figure 3A, Supplementary Table S3). At the lowest concentration, 0.07 μL/cm^2^, significantly high mortality was observed only after the use of the *A. sativum* EO (Figure 4A, Supplementary Table S3).

*Tribolium confusum*

Figure 2B and Supplementary Table S3 show the response of the *T. confusum* after the application of nine repellents at a concentration of 3.7 μL/cm^2^. The DEET and the EO of *A. annua* had minimal effects at 24, 48, and 72 h after treatment. By contrast, very high mortality was caused by the 2-undecanone. The essential oils of *A. sativum*, *O. basilicum, C. longa, E. globulus*, and *L. augustifolia* were found to be particularly lethal, as mortality reached 90%. The use of the EOs from *A. sativum*, *O. basilicum*, *C. longa*, and *E. globulus* at a concentration of 0.37 μL/cm^2^ caused extensive deaths of the adult *T. confusum*. By contrast, the EO of *A. annua* did not cause mortality. The remaining repellent substances significantly affected mortality after 24 h, except for the DEET, which did not show an effect until 72 h after treatment (Figure 3B, Supplementary Table S3). The lowest concentration, 0.07 μL/cm^2^, was lethal to 100% of the beetles tested after 24 h when the EOs from *O. basilicum, A. sativum,* and *E. globulus* were used. After the 48- and 72-h treatments, a significant mortality effect was also observed from the DEET, 2-undecanon, and EOs of *L. angustifolia*, *P. sylvestris*, and *C. longa* (Figure 4B, Supplementary Table S3).

*Acanthoscelides obtectus*

At a concentration of 3.7 μL/cm^2^, all the repellents, except the *A. annua* EO, caused the mortality of *A. obtectus*. The highest mortality was caused by the 2-undecanone and the EOs of *A. sativum*, *L. augustifolia*, *E. globulus*, and *O. basilicum* (Figure 2C, Supplementary Table S3). A similar result was also obtained after the application of repellents at a concentration of 0.37 μL/cm^2^, except for the maximum effect of the *L. augustifolia* (Figure 3C, Supplementary Table S3). At a concentration of 0.07 μL/cm^2^, the high toxicity of the 2-undecanone and the EO of *A*. *sativum* was confirmed at all the time points. Moreover, the EOs of the *E. globulus* and *O. basilicum* completely decreased the population after 72 h (Figure 4C, Supplementary Table S3). In general, the essential oils of the *A. sativum*, *E. globulus*, and *O. basilicum* had the highest mortality effect, while the mortality effect of the *L. augustifolia* EO decreased in line with the concentration.

## 3. Discussion

Currently, synthetic pesticides are still used to control stored-product pests. Synthetic pesticides are widely used in traditional, developing, and emerging countries [28]. With regard to the environment and the threat of rapidly growing resistance, there is an urgent need to identify new and safer options [29].

Recently, the use of repellents has attracted the interest of scientists and the crop-protection industry [30]. The most promising are DEET and 2-undecanone, which are already used as components of insect repellents [31]. A significant effect of DEET is well described for *Lasioderma serricorne*, *Rhyzoptera dominica*, *Liposcelis bostrychophila*, and *Tribolium castaneum*, for which two or three hours of treatment caused high mortality (78%, 94%, 96%, and 92%, respectively) in low concentrations [32,33]. However, in our experiment, the mortality increased in line with the concentration and the overall effect on the mortality rate was negligible for all the pests, contradicting the reports in previous studies. By contrast, the repellence effect of the 1% solution was surprisingly high (>97.5%). A similar trend was described by Brown and Hebert [34], who revealed the direct influence of DEET repellence and used the concentration on the most common mosquitoes, *Aedes aegypti* and *Culex pipiens*. However, they stated that a higher DEET content might affect the quality of impregnated goods and might extend the effect only by an hour. Further, 2-undecanone showed a significant repellence effect (78.5%) on *Ahasverus advena* [35]. Our olfactory test confirmed these results, as using 1% solution caused 100% repellence in *T. molitor*, *T. confusum*, and *A. obtectus*. The 2-undecanone showed an eliminating effect on *A. obtectus* in all the used concentrations; however, the other tested pests showed high survival, even after the highest concentrations were used. Interestingly, 2-undecanone is known to activate and inhibit mosquitos’ olfactory receptors. Therefore, its activity is modulated, which can have an agonistic or antagonistic effect [36]. This discovery might help to exploit 2-undecanone in developing biodegradable alternatives for synthetic pesticides against pests. Additionally, an antifeedant effect of 2-undecanone against *Spodoptera frutiperda* larvae was observed by Ayil-Gutiérrez et al. [37]. From the accessible results, we conclude that both chemicals might work more as preventative tools with a repellence effect rather than in the elimination already infected storages.

The other promising choices might be natural volatile extracts, as essential oils feature repellent and biological activity [29]. The chemical profiles of the EOs used in this study were analyzed, considering the dissimilarities between the chemical profiles according to geographical origin [24,38]. After comparing the chemical profiles used in our study with those in previous reports [16,39,40], no differences were revealed.

*Allium sativum* extracts have substantial acaricidal and insecticidal properties against coleopteran and dipteran pests [41]. As with the findings in [42,43,44], our results confirmed a high repellence ability (>90%) against *Tenebrio molitor* and a high affinity with *A. obtectus* and *T. confusum*. Strikingly, s high efficiency (more than 95%) was detected even in the lowest concentration (0.01%). Our data showed high toxicity (more than 95%) after using the EOs of *Allium sativum*. These results were condensed in [43,45], in which 50% mortality was reached in *T. castaneum* and *Ephestia kuehniella* after 24 h of treatment. Interestingly, the lethal effect decreased synergistically in line with the concentration. This pattern was also described in previous studies (i.e., [10,42,46,47]). Moreover, it seems that *A. sativum* extract has an inhibition effect on larvae development [48]. Combined with its long-lasting protection period of up to 135 days [10] and only negligible damage to stored crops [49], this means that EO appears to be the perfect tool for pest management.

The high toxicity to our pests was caused by EO extracted from *O. basilicum* – with a mortality rate > 98 % for *T. confusum* and *A. obtectus* and moderate for *T. molitor*. Our results are in accordance with those of Rodríguez-González et al. [50], who reported 33% mortality in 24–72 h experiments on *A. obtectus*. Furthermore, our experiments on *T. molitor* confirmed the synergistic effect of increasing effectiveness over time, previously described by Rodríguez-González et al. [50]. In addition, the repellence effect remained conspicuous, even though low concentrations were used. 

The *Eucalyptus globulus* EO had a noticeable effect on the *T. confusum* and *A. obtectus*, which corresponded to the mortality previously obtained for *E. kuehniella* (80% within 24 h) [45]. Even though our mortality results for *T. molitor* were moderately low, the similarity to the results previously obtained for *T. castaneum* [51] is obvious. However, the *A. obtectus* mortality rate differed from those observed by Bittner et al. [52] and Papachristos et al. [53]. The higher concentration of EO used in our experiments may have been the cause. 

The third EO to have previously shown promising toxicity results on *T. confusum* and *A. obtectus* was from *L. angustifolia.* Our pests, *A. obtectus*, *Rhyzoptera dominica* and *Sitophilus granaries*, also suffered from high mortality rates at low concentrations of *L. angustifolia* EOs (50% within 24 h) [53,54,55]. Interestingly, Germinara et al. [54] noted that the mortality effect of the EO might decrease by up to 10 times when the EO is used on infected grains. Furthermore, the repellence activity of *L. angustifolia* EO seems to be high (>80%) in all the studied pests. The effect was recognizable even at low concentrations, which was consistent with the findings on *S. granaries* [54]. 

Conversely, the lowest effect on our pests was observed in the extracts from *C. longa* and *A. annua*. In the case of *C. longa*, the mortality tests showed high toxicity for *T. confusum* and *A. obtectus*. By contrast, *T. minor*’s mortality reached only about 10%. This result supports the low efficiency of *Tribolium castaneum* adults reported by Ali et al. [48]. Surprisingly, no mortality caused by the *A. annua* EO was observed. This was inconsistent with the results obtained for all the adult pests of *Solenopsis invicta* and *Callosobruchus maculatus* [56,57]. *C. longa* and *A. annua*’s significant repellence effects were recognized in all the tested pests, as in [7]. However, the effect of the low EO concentrations is noteworthy. Even though our results showed minimal effects, their potential use might reside in their inhibition of egg hatchability and the reduction in pests’ developmental rate, as reported in [7].

Generally, in all our pests, the mortality increased over time and in line with the concentrations of the EOs. Furthermore, our tests suggested that eucalyptol, limonene, and O-cymene dispose of pests with high toxicity. In addition, the repellence effect was also significant, as is also acknowledged by different studies using various EOs [29,58,59,60,61,62,63]. Hence, we can conclude that these EOs might have significant potential against an extensive range of pests.

To summarize, synthetic insecticides kill target insect pests quickly and provide excellent control when applied. However, their applications leave toxic residues in stored grains, which are harmful to consumers. Therefore, a new and valuable alternative approach to reducing the use of insecticides in warehouses on stored grains should be found. One potential method uses the encapsulation and emulation of botanical EOs as surfactants in greenhouses [64]. The second is the direct application of EOs. Both approaches might be powerful tools for integrated storage-pest management. However, more research needs to be conducted in the laboratory and also in the field.

## 4. Materials and Methods

### 4.1. Storage Pests 

*Tribolium confusum, Tenebrio molitor,* and *Acanthoscelides obtectus* were used in laboratory experiments and olfactometric tests with repellents. They were grown under controlled conditions at the Department of Plant Protection, Slovak University of Agriculture, Nitra, Slovakia. The culture medium consisted of 1000 g of whole-grain flour from Pohronský Ruskov a.s., 500 g of oat flakes from Štúrovo flour mill, and 20 g of dry yeast from AGROFORTEL, s.r.o. In the case of the bean weevil, the medium consisted of 100% beans (*Phaseolus coccineus*) of the Lady Di variety from Semena Online, s.r.o. The medium was mixed by hand and stored at a temperature of 25 °C for 24 h. Healthy beans were stored at 25 °C for 24 h after beetle release. Approximately 400 adult beetles were stored at 25 ± 2 °C and 50 ± 5% relative humidity at a 16:8 long-day cycle in plastic boxes (37 cm × 26 cm × 14 cm). Both sexes and approximately equal ages were used in the experiments.

### 4.2. Essential Oils and Chemical Substances 

The oils were extracted as commercial essential oils by Mystic Moments (2020) Inc. Hampshire, (UK) and Forest Centre Herbs (2019) St. Louis, MO, (USA) from plant species from the families Amaryllidaceae (*Allium sativum*), Myrtaceae (*Eucalyptus globulus*), Lamiaceae (*Ocimum basilicum*, *Lavandula angustifolia*), Zingiberaceae (*Curcuma longa*), Asteraceae (*Artemisia annua*), and Pinaceae (*Pinus sylvestris*). Many foods are susceptible to microbial spoilage, with contamination involving high water activity. In addition, essential oils have low water solubility [65]. Therefore, the emulsifiers were used as antimicrobial agents. TWEEN 80 is a well-known food-grade emulsifier that is commonly used for this purpose [66]. The chemical substances DEET (N,N-diethyl-3-methylbenzamide; purity 97%, CAS 134-62-3) and 2-undecanone (methyl nonyl ketone; purity 99%, CAS 112-12-9) were obtained from Sigma-Aldrich (St. Louis, MO, USA) (2019).

### 4.3. Identification of Chemical Components by Gas Chromatography–Mass Spectrometry Analysis (GC/MS) 

Prior to the experiment, the chemical constituents of plant EOs were identified by gas chromatography–mass spectrometry (GC/MS) analysis using GC Agilent 7890B and MS Agilent 5977A (Agilent Technologies Inc, Santa Clara, CA, USA at AgroBioTech at the Slovak University of Agriculture in Nitra. Essential oil samples were diluted in hexane (HPLC ≥ 97%, Sigma Aldrich GmbH, Darmstadt, Germany) at a concentration of 10 μL/mL. One microliter of the diluted sample was injected into the inlet (250 °C), which was operated in 1:10 split mode. Separation was performed using a HP-5 ms capillary column (30-m × 0.25-mm × 0.25-μm film; Agilent Technologies). The oven temperature was set at 50 °C for the first 5 min and then increased at a rate of 3 °C/min to 240 °C, after which it was held constant for 2 min. Helium was used as the carrier gas at a constant flow rate (1.2 mL/min). The mass detector had a filament-ionization energy of 70 eV, a transfer-line temperature of 250 °C, a MS source temperature of 230 °C, and a quadrupole temperature of 150 °C. The mass spectrometer was programmed under electron impact (EI) in full scan at m/z 40–350 with a scan rate of 2.4 scans/s. Compounds were identified by comparing the mass spectra (over 80% agreement) with a commercial database, NIST 2017, and the Wiley library of retention times of reference standards to compare occurrence data in EOs with those in the literature [67].

### 4.4. Olfactometer Studies 

The behavior of stored-product pests used for repellent trials was examined using a Y-shaped glass-tube olfactometer modified from Sigma Scientific LLC of Florida (Micanopy, FL, USA). The Y-tube system included separate secondary filter cartridges for each channel of the system, a glass-threaded drain chamber with air-odor bypass, an air-supply system, and a vacuum-draw system. The inner diameter of the Y-pipe arms was 4 cm, the arm length was 10 cm, and the shaft length was 5 cm. The acute angle between the Y-tube and the horizontal plane was 45°. Ten beetles were released within the first centimeter of the base tube of the olfactometer. They ran up the tube. When the air supply was turned on, the recording of the experimental time began. It controlled the flow rate (volume) of air passing through the cabin arms of the Y-tube. The air was refuelled with a standard compressor and filtered with an air-delivery system. The total air flow for each chamber was 125 mL per minute. Arriving at the Y-junction, the beetles chose between the clean and the odor-laden airflow. Each beetle tested was considered to have made a choice if it moved halfway through the Y-tube arm toward the odor sources. If the beetles did not choose an arm within 10 min, a zero was recorded. The average duration of replicates was 10 min. The beetles that entered the arm, connected with the odor, and stayed for at least 10 s were recorded as attracted. The insects that entered the arm with the control and stayed for at least 10 min were recorded as repelled. Each experiment was performed in six replicates. For each bioassay, 100-milligram cotton swabs were dosed with 5 μL of the treatment solution and placed in one of the chambers of the olfactometer. The other arm received 5 μL TWEEN 80 as a control. Chemicals (DEET, 2-undecanone) and EOs (*A. sativum, E. globulus, O. basilicum, C. longa, A. annua, P. sylvestris*, and *L. angustifolia*) were prepared at three concentrations (1%, 0.1%, and 0.01%). The experiment in a Y-tube was conducted in a laboratory at a temperature of 25 °C and a relative humidity of 30%. After completion of the experiments, the entire Y-tube was cleaned with hot soapy water, wiped, and dried at the end of the day.

### 4.5. Evaluation of Toxicity

The Petri-dish method was used to evaluate toxicity. The filter paper was placed on the bottom of the petri dish (90 × 15 mm) (60 mm diameter, Whatman No.1). Adults of *T. confusum*, *T. molitor* and *A. obtectus* were used for the experiment. In the center of each Petri dish, one of the chemicals or EOs (doses of 3.7, 0.37, 0.07 L/cm^2^) enriched with a surface agent TWEEN 80 was applied [68,69]. TWEEN 80 (5 µL) was used as a control treatment. Six replicates of each treatment were performed. After evaporation (1 h), ten adults were placed in the center of the arena for each concentration and for the control. Adult mortality was observed and counted every 24, 48, and 72 h. Adults were considered dead if they did not move. 

### 4.6. Analysis of the Data

Results were recorded using Prism graphing software (GraphPad Software 6, San Diego, CA, USA). One-way ANOVA and Dunnett’s multiple-comparison test were performed.

## 5. Conclusions

The repellent or toxic effects of the essential oils in this study were not based on specific chemicals common to all the essential oils. The essential oils tested had an unfavorable effect on the three storage pests—*Tribolium confusum*, *Tenebrio molitor*, and *Acanthoscelides obtectus*. Our results indicate that *A. sativum* and *O. basilicum* were the most effective against *T. castaneum* and *T. molitor*; at the same time, *L. angustifolia* and *C. longa* showed high activity against *A. obtectus*. We also found that the most effective EOs still exhibited remarkable repellent and mortality activity when diluted to low concentrations (0.01%, 0.07 μL/cm^2^). These EOs can be proposed as sufficient biological-control agents in different storage conditions.

## Data Availability

All published data are available within the article and Supplementary Materials.

## Supplementary Materials

The following supporting information can be downloaded at https://www.mdpi.com/article/10.3390/plants11223077/s1. Table S1: The results of gas chromatography–mass spectrometry (GC–MS) analysis of selected EOs. Compounds whose content exceeded 0.1% in tested essential oils are listed. Table S2: The results of statistical tests of two chemical substances, seven essential oils (EOs), and three warehouse pests, *Tenebrio molitor*, *Tribolium confusum*, and *Acanthoscelides obtectus* to confirm the repellent effect. Statistically significant *p* values are marked with an asterisk. Dunnett’s multiple-comparison tests were used. Table S3: The results of statistical tests of two chemical substances and seven essential oils (EOs) and three warehouse pests, *Tenebrio molitor*, *Tribolium confusum*, and *Acanthoscelides obtectus* to confirm the mortality. Statistically significant *p* values are marked with an asterisk. Dunnett’s multiple-comparison tests were used.
